# Increasing the sensor channels: a solution for the pressing offsets that cause the physiological parameter inaccuracy in radial artery pulse signal acquisition

**DOI:** 10.3389/fbioe.2024.1359297

**Published:** 2024-02-15

**Authors:** Chao Chen, Zhendong Chen, Hongmiin Luo, Bo Peng, Yinan Hao, Xiaohua Xie, Haiqing Xie, Xinxin Li

**Affiliations:** ^1^ School of Computer Science and Engineering, Sun Yat-Sen University, Guangzhou, China; ^2^ Science and Technology Innovation Center, Guangzhou University of Chinese Medicine, Guangzhou, China; ^3^ Department of Musical Instrument Engineering, Xinghai Conservatory of Music, Guangzhou, China; ^4^ Sniow Research and Development Laboratory, Foshan, China; ^5^ School of Medical Engineering, Foshan University, Foshan, China; ^6^ School of Nursing, Sun Yat-Sen University, Guangzhou, China

**Keywords:** multi-channel pulse signals, tactile sensors, tidal peak, pulse wave analysis, biomedical engineering

## Abstract

**Introduction:** In studies of pulse wave analysis, single-channel sensors only adopt single temporal pulse signals without spatial information to show pulse-feeling patterns. Multi-channel arterial pulse signals, also named as three-dimensional pulse images (3DPIs), provide the spatial and temporal characteristics of radial pulse signals. When involving single or few-channel sensors, pressing offsets have substantial impacts on obtaining inaccurate physiological parameters like tidal peak (P_2_).

**Methods:** This study discovers the pressing offsets in multi-channel pulse signals and analyzes the relationship between the pressing offsets and time of P2 (T_2_) by qualifying the pressing offsets. First, we employ a data acquisition system to capture 3DPIs. Subsequently, the errorT_2_ is developed to qualify the pressing offsets.

**Results:** The outcomes display a central low and peripheral high pattern. Additionally, the errorT_2_ increase as the distances from the artery increase, particularly at the radial ends of the blood flow direction. For every 1 mm increase in distances between sensing elements and center sensing elements, the errorT_2_ in the radial direction escalates by 4.87%. When the distance is greater than 3.42 mm, the errorT_2_ experiences a sudden increase.

**Discussion:** The results show that increasing the sensor channels can overcome the pressing offsets in radial pulse signal acquisition.

## 1 Introduction

**FIGURE 1:**
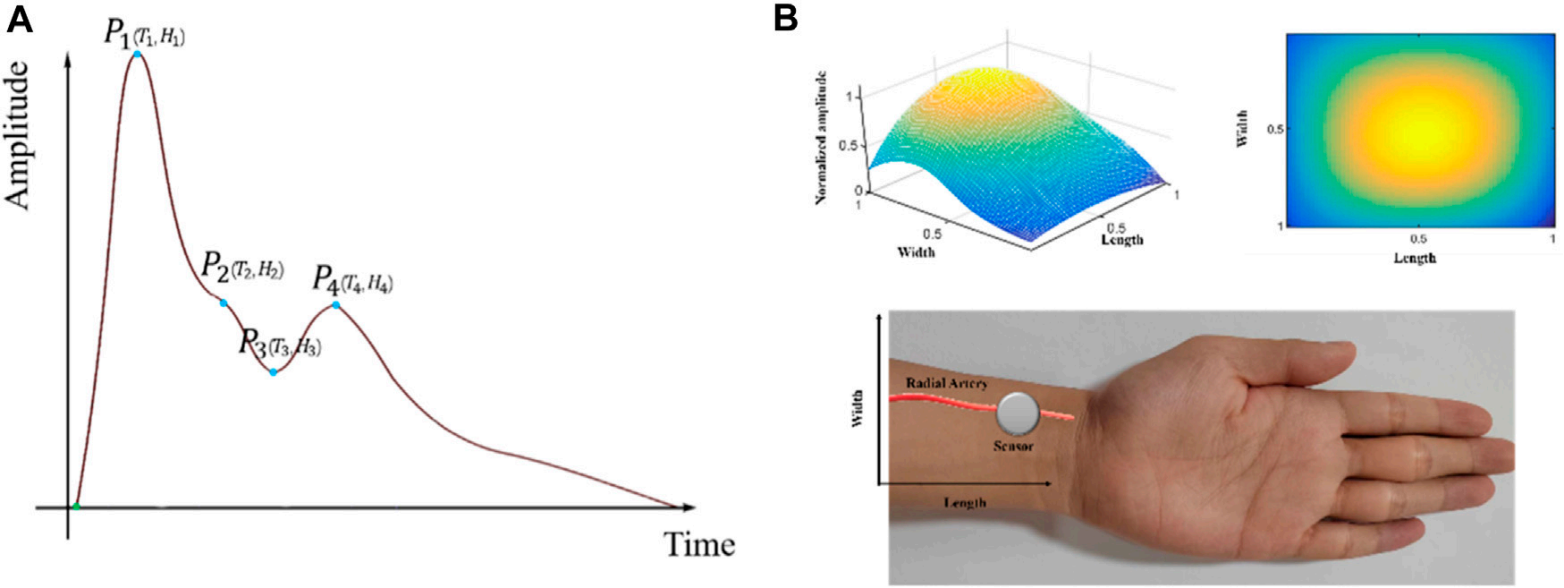
3DPIs during a cardiac cycle: **(A)** One-dimensional radial arterial pulse wave and four key physiological points; **(B)** Stereoscopic (upper left side) and planar (upper right side) 3DPIs at the T_2_ with normalized sensing length, width, and pulse amplitude; data acquisition by a sensor from a radial artery (lower side).

Cardiovascular diseases (CVD) are the primary causes of death globally (Visseren et al., 2021). Pulse waves are generated by cardiac ejection during heart cycles and play a significant role in cardiovascular health. Pulse wave analysis (PWA), considered an essential diagnostic tool for assessing cardiovascular disease, detects early pathological modifications, such as arterial stiffness and endothelial dysfunction (AlGhatrif et al., 2013; Matsuzawa et al., 2015; Francque et al., 2016; Ohkuma et al., 2017; Chen et al., 2021). The roles of PWA in the diagnosis of CVDs encompass the assessment of arterial stiffness, pulse wave velocity analysis, and pulse waveform analysis. Several physiological parameters, such as the peripheral augmentation index (pAIx), pulse transit time (PTT), reflection magnitude (RM), and reflection index (RI), are employed in PWA (Munir et al., 2008; Wang et al., 2010; Milicevic et al., 2020; Yao et al., 2022; Campitelli et al., 2023). The tidal peak (P_2_), representing the second wave peak of the pure radial arterial pulse signals within a cardiac cycle, is one of the four key physiological points, which also include percussion peaks (P_1_), diastolic notches (P_3_), and diastolic peaks (P_4_) (Su et al., 2016). These points represent key temporal markers within each cardiac cycle, conveying essential information about cardiovascular health. The P_2_ reflects the pressure wave originating from peripheral resistance vessels, which reflects the amplitudes of arterial stiffness and is crucial in the assessment of arterial stiffness (Peng et al., 2022; Chen et al., 2023). Figure 1A illustrates the positions of the four key physiological points during a cardiac cycle.

To measure radial pulse signals, a series of acquisition devices have been developed and applied in pulse acquisition (Luo et al., 2012a; Qiao et al., 2018; Tsai et al., 2018; Fu et al., 2019; Huang et al., 2019). These devices employ sensors with one sensing element to capture radial pulse signals. However, these single-channel sensors only adopt single temporal pulse signals without spatial information to show pulse-feeling patterns (Kim et al., 2011; Kim et al., 2012; Huang et al., 2019). In recent years, multi-channel pulse signals have been widely measured using tactile sensors for pulse signal application studies (Chung et al., 2011; Kong et al., 2016; Peng et al., 2019; Zhang et al., 2021; Liu et al., 2023). The multi-channel arterial pulse signals, also named as three-dimensional pulse images (3DPIs), provide the three-dimensional spatial and temporal characteristics of pulse signals (Peng et al., 2019). 3DPIs also enhances the visualization of pulse signals and provides more cardiovascular-related information. During data acquisition, 3DPIs at T_2_ (time of P_2_) are often focal points of interest (Cui et al., 2019; Yuen et al., 2019; Luo et al., 2021). Figure 1B displays the stereoscopic/planar 3DPIs at the T_2_ and data acquisition by a sensor from a radial artery. Most devices for obtaining 3DPIs fix the tactile sensors, which are a kind of multi-channel sensors, on the robotic finger and control the robotic finger to press on the position of radial arteries to acquire the pulse signals (Chung et al., 2011; Si et al., 2011; YF et al., 2011; Luo et al., 2012a; Luo et al., 2012b; Chung et al., 2012; Hu et al., 2012; Chung et al., 2013; Luo and Chung, 2016; Luo et al., 2016; Luo et al., 2018; Peng et al., 2019).

During the pressing process, the T_2_ are different in all channels of the tactile sensors (Peng et al., 2019). However, in scenarios involving sensors with one or few sensing elements, pressing offsets are inevitable. In such cases, the operators may face difficulties in judging whether the pressing process is accompanied by offsets since the sensitivities of single-channel sensors are not uniform (Dario and Bergamasco, 1988; Dario and Buttazzo, 2016; Fearing, 2016). Subsequently, operators may encounter challenges in obtaining multi-channel pulse signals that reflect true arterial conditions. Then, operators may obtain inaccurate physiological parameters like T_2_. Operators encounter a challenge when detecting pressing offsets during the acquisition of multi-channel pulse signals due to the lack of appropriate quantitative tools for this purpose. By detecting whether any offsets occur during the pressing process, operators can adjust the pressing position to ensure the acquisition of 3DPIs that accurately reflect the arterial conditions.

This study discovers the pressing offsets in multi-channel pulse signals and analyzes the relationship between the pressing offsets and T_2_ by qualifying the pressing offsets in pulse signal acquisition. First, we employ a data acquisition system to capture 3DPIs from the subjects. Then, the errors between each channel and the best channel in 3DPIs are determined to qualify the pressing offsets in the tactile sensor. Finally, the subjects are divided into multiple control groups, and the results are compared and analyzed. Due to the high sensitivity and mature fabric technologies, operators prefer to employ tactile sensors to obtain pulse signals (Yang et al., 2017). In this study, we employ the tactile sensors developed by PPS (Pressure Profile Systems Inc., Los Angeles, CA, United States), which are widely recognized as the preferred choice for acquiring physiological signals across a diverse range of clinical environments (Cui et al., 2019). PPS tactile sensors are designed with exceptional sensitivity and accuracy to detect tiny changes in pressure that are indicative of pulse signals. Electrodes as sensing elements are arranged in orthogonal, overlapping strips to create a tactile sensor. The electrodes overlap at each position to form a discrete capacitor. A single row and column are selectively scanned to determine the capacitance and pressure (Hu et al., 2012). PPS sensors offer the ability to customize tactile sensors to meet specific research or clinical needs, thus PPS sensors come in a variety of specifications. In this article, a PPS sensor with 5 rows and 5 columns is denoted as a 5 × 5 sensor. The 5 × 5–1 PPS sensor can provide a tactile center point by positioning the blank area in the corner (Peng et al., 2019).

The rest of this paper is organized as follows. In Methods section, data construction, preprocessing, and pressing offset evaluation are introduced. The Results section illustrates the results of different control groups. Lastly, the final section concludes with a discussion of the experimental results.

## 2 Materials and methods

**FIGURE 2:**
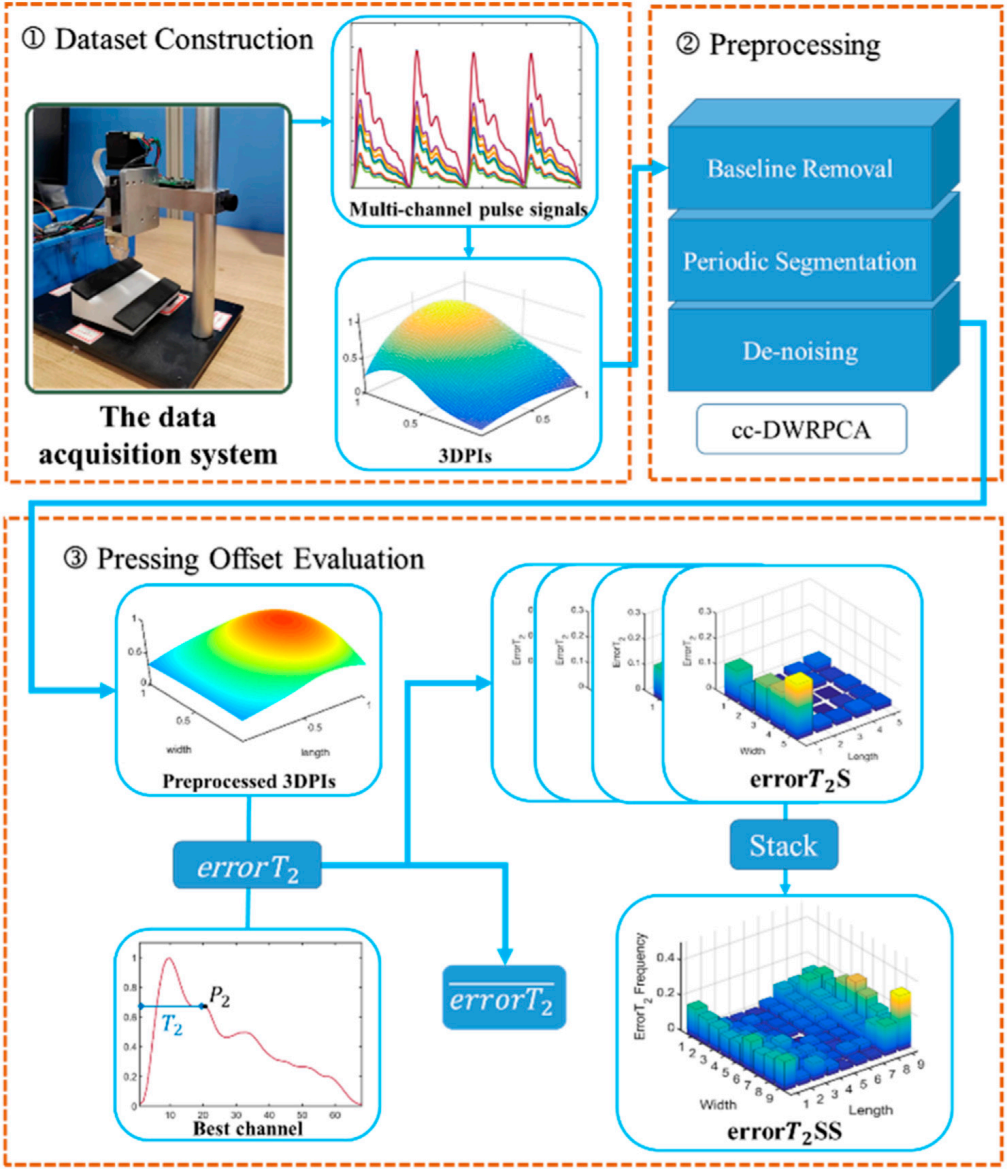
The flow chart of the study.

Figure 2 presents a comprehensive depiction of the entire workflow, consisting of three primary stages: dataset construction, preprocessing, and pressing offset evaluation. The dataset construction section involves utilizing a multi-channel pulse acquisition device to capture pulse signals from the subjects. Subsequently, the acquired pulse wave dataset undergoes preprocessing, which includes baseline removal, periodic segmentation, and de-noising. Finally, the preprocessed data is then utilized for calculating the $\bar{errorT_{2}}$ and facilitating the construction of errorT_2_SS.

### 2.1 Dataset construction

#### 2.1.1 Data acquisition

**FIGURE 3:**
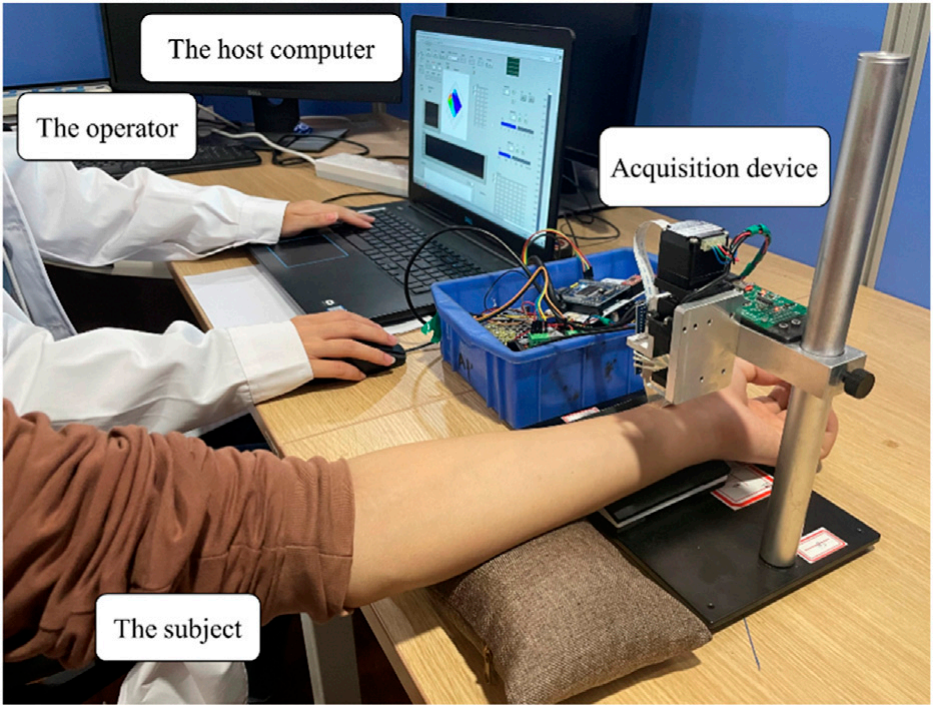
The data acquisition system in this study.

This study employs a data acquisition system for capturing pulse signals from subjects. In the data acquisition system of this study, we employ two strategies to ensure the accurate acquisition of 3DPIs. We utilize a specialized pulse signal acquisition device, which include PPS sensors, the most popular tactile sensors, and follow stringent data collection protocols. A PPS sensor, a fixture, a robotic finger, a stepper motor (to control the pressing depth of the robotic finger), and a main control board make up the uniaxial device (Figure 3). This study utilizes a PPS tactile sensor with an 85 Hz sampling rate. The rectangular geometry of a sensing element in the PPS tactile sensor is 1.25 × 1.71 mm (Peng et al., 2019). The main control board, based on STM32 (Jin et al., 2019), is responsible for managing the underlying hardware, collecting PPS data, and establishing serial port communication with the host computer. Pulse signals are detected using a 24-channel PPS tactile sensor. Subjects are scheduled to sit in a chair in a quiet room for a minimum of 10 min before their pulse signals are recorded. The operator can easily handle the computer and oversee the measurement process while the subject is comfortably tested on their wrist at the same height as their heart due to their proper relative locations. The data collection process adheres strictly to the protocol established in our previous research (Peng et al., 2019).

#### 2.1.2 Subjects and control groups

The institutional review board at the National Cheng Kung University Hospital granted authority for this study to conduct a human trial (Approval Number: B-ER-103-263). The collected personal data from 52 subjects, with sequentially acquired valid data for both hands, is examined and analyzed (Table 1). Among the subjects, 15 are non-hypertensive subjects, and 37 are hypertensive patients. Finally, 623 multicycle pulse signal samples with 24 channels (5 × 5–1) are acquired. In this study, the subjects are divided into three control groups: left hands/right hands, male/female, and non-hypertensive/hypertensive.

**TABLE 1:** Subject characteristics in this study (Mean ± Standard Deviation).

	All	Male	Female
Number	52	44	8
Age (year)	59.98 ± 14.94	59.64 ± 15.47	61.88 ± 15.47
Height (cm)	164.25 ± 7.48	165.59 ± 6.673	156.88 ± 6.67
Weight (kg)	72.17 ± 12.91	72.45 ± 12.97	70.63 ± 12.97
BMI (kg/m^2^)	26.68 ± 4.00	26.32 ± 3.77	28.67 ± 3.77
Systolic BP (mmHg)	136.96 ± 23.59	135.70 ± 21.25	143.88 ± 21.25
Diastolic BP(mmHg)	80.17 ± 14.03	81.20 ± 13.70	74.50 ± 13.70
Heart rate (beats/min)	69.54 ± 10.30	70.39 ± 9.12	64.88 ± 9.12

### 2.2 Preprocessing

Numerous studies have employed a thorough and suggested pulse signal pipeline to preprocess pulse signals (Wang et al., 2016; Peng et al., 2019; Peng et al., 2022; Song et al., 2023). In this section, a complete preprocessing pipeline includes baseline removal, periodic segmentation, and de-noising.

First, to eliminate baseline wandering from the signals, a first-order Butterworth high-pass filter with a cut-off frequency of 0.5 Hz is applied (Li, 2007). Second, the multi-channel signals are segmented into individual single-period pulse signals for the de-noising demand of the subsequent algorithm. Finally, a de-noising algorithm called Cross-Channel Dynamic Weighting Principal Component Analysis (cc-DWRPCA) is implemented to de-noise the processed pulse signals (Peng et al., 2022).

The cc-DWRPCA, based on Robust Principal Component Analysis (RPCA) (Candès et al., 2011), employs a channel-scaled factor (CSF) technique to manipulate the weights ω among channels (see Eq. 1), which can get superior performances than weighted robust principal component analysis (WRPCA) (He et al., 2019). Without using the inherent correlations between these channels, WRPCA is able to extract signal properties for each channel independently. After de-noising with cc-DWRPCA, 623 single-cycle pulse signals with 5 × 5–1 are extracted.

$$\omega_{C}=\frac{\eta }{\sigma_{C}\left(D\right)+\varepsilon },$$ (1)

where $\omega_{C}$ is the weight of the *c*th channel of a subject, η denotes the *c*th CSF of D in the c -th channel, $\sigma_{C}\left(D\right)$ represents *c*th singular value of *D*, *D* is the singular value decomposition of a multicycle pulse signal, ε is the reconstruction error tolerance, which is set to 1e-6 in this study.

To obtain a complete 5 × 5 matrix, we employed interpolation to generate an additional channel for each sample, transforming the 5 × 5–1 matrix (24 channels) into a 5 × 5 matrix (25 channels). Consequently, a complete 5 × 5 3DPI is available for each sample. Eq. 2 shows the arrangement of a 5 × 5 tactile sensor.

$$\left(\begin{array}{ccccc} 1 & 6 & 11 & 16 & 21\\ 2 & 7 & 12 & 17 & 22\\ 3 & 8 & 13 & 18 & 23\\ 4 & 9 & 14 & 19 & 24\\ 5 & 10 & 15 & 20 & 25 \end{array}\right),$$ (2)

where the numbers in the matrix represent the numbers of channels.

### 2.3 Pressing offset evaluation

In this article, the errors between each channel and the best channel in 3DPIs are determined to evaluate the pressing offsets. Following preprocessing, channels with the maximum amplitude are automatically selected as the best channels for each sample. Using these best channels as a benchmark, a stringent manual screening of the samples is conducted by a cardiovascular expert, who also provided manual annotations for the P_2_ position. The expert annotation serves as the gold standard for P_2_ localization. In this section, errorT_2_, the average errorT_2_ ( $\bar{errorT_{2}}$ ), and errorT_2_ Stacked Surface (errorT_2_SS) are proposed to evaluate the pressing offsets.

By performing manual annotations for P_2_, the T_2_ across all channels are obtained for each sample. Subsequently, for each sample, the error between each channel and the maximum channel, representing the errorT_2_ for each channel, are calculated (see Eq. 3). Then, $\bar{errorT_{2}}$ across all channels for all subjects are calculated (see Eq. 4). Based on the specifications of the 5 × 5 tactile sensors, we arrange the errorT_2_ of 25 channels and ultimately obtain the errorT_2_ Surface (errorT_2_S) matrix for each sample. The errorT_2_ can assess the discrepancy between T_2_ of a single channel and the best channel, while errorT_2_S evaluates the pressing offset for a sample. ErrorT_2_S can also assess the discrepancy between T_2_ of each channel and the best channel in the spatial domain, thereby facilitating an understanding of the distribution of pulse extraction center points.

$$errorT_{2}\left.\left(n,c\right)=\right|{T_{2}}_{C}\left(n\right)-{T_{2}}_{best}\left.\left(n\right)\right|,$$ (3)

where *c* represents the number the channel, ${T_{2}}_{C}\left(n\right)$ is T_2_ in the *c*th channel of sample *n*, ${T_{2}}_{best}\left(n\right)$ is T_2_ in the best channel of sample *n*.

$$\bar{errorT_{2}}\left(n\right)=\frac{\sum_{c}^{\Gamma }\left|{T_{2}}_{C}\left(n\right)-{T_{2}}_{best}\left.\left(n\right)\right|\right.}{\Gamma },$$ (4)

where Γ is 25, the number of all channels in this study.

**FIGURE 4:**
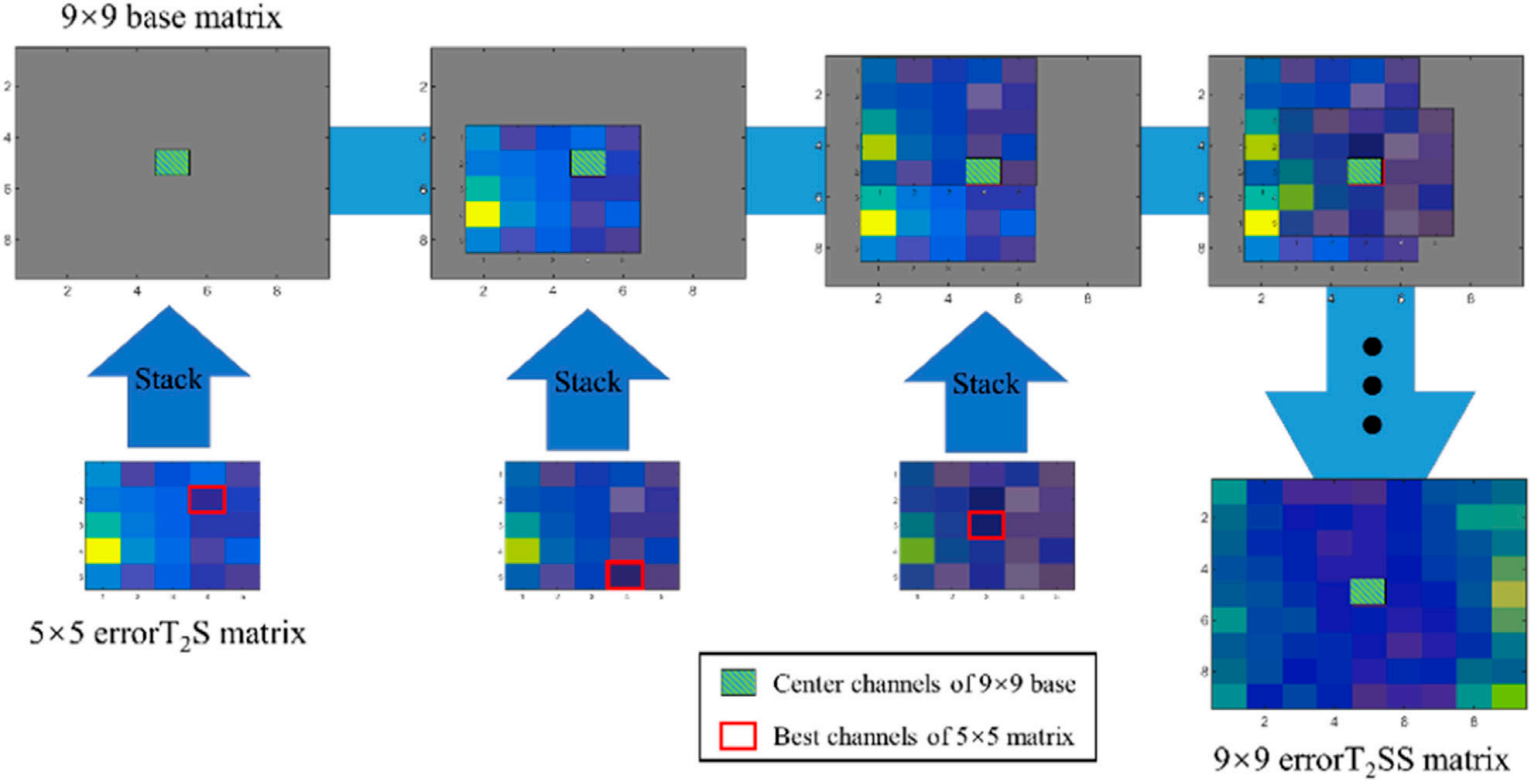
The stacking process of the errorT_2_SS.

To investigate the offset degree of the robotic finger pressing process during data collection, the errorT_2_S of all samples are stacked to construct the errorT_2_SS. Algorithm 1; Figure 4 demonstrate the construction process of the errorT_2_SS. Firstly, a 9 × 9 zero matrix is created to represent the 9 × 9 channel matrix as the base. Subsequently, the best channels of each errorT_2_S are aligned with the center channel of the base for stacking. Lastly, the weighted errorT_2_ are calculated for each channel within the 9 × 9 base (see Eqs 5, 6). The obtained weighted errorT_2_ constitute the errorT_2_SS matrix.

$$Weighted\;errorT_{2}\left(c\right)=\sum_{n}^{N}\omega_{n}∙errorT_{2}\left(n,c\right),$$ (5)

$\omega_{n}$ represents the weight in *c*th channel, which is defined as:

$$\omega_{n}=\left\{\begin{array}{c} 1,{errorT_{2}\left(n,c\right)}_{9\times 9}>0\\ 0,{errorT_{2}\left(n,c\right)}_{9\times 9}=0 \end{array}\right.,$$ (6)

where ${errorT_{2}\left(n,c\right)}_{9\times 9}$ denotes the *c*th $errorT_{2}$ that errorT_2_S of a sample is stacked and aligned to the center channel of the 9 × 9 base.

Algorithm 1 Construction process of the errorT_2_SS.

 **Input**: T_2_: time of P_2_;
 S: pulse signals, S_C_ is the pulse signal of the *c*th channel;
 N: number of samples, n is the *n*th sample;
 M: Arrangement of the 5 × 5 tactile sensor
 **Output**: errorT_2_SS: errorT_2_ Stacked Surface matrix
1:  Initialize an empty errorT_2_ Surface (errorT_2_S) matrix
2:  **for** each sample **in** *n* **do**
3:   extract channel with maximum amplitude as *S*_*best*_ *(n)*
4:   for each channel in all channels of sample *n* do
5:   calculate errorT_2_ for *S*_*C*_ *(n)* using Eq. 3
6:   **end for**
7:   arrange all errorT_2_(*c*) of 25 channels according to M to form errorT_2_S (*n*)
8:  **end for**
9:  Initialize a 9 × 9 zero matrix as base
10:  **for** each errorT_2_S(*n*) **do**
11:   align best channels with the center channel of the base
12:   stack the aligned errorT_2_S(*n*) on top of the base
13:  **end for**
14:  calculate the weighted errorT_2_ for each channel using Eq. 5
15:  form errorT_2_SS matrix using the weighted errorT_2_

## 3 Results

### 3.1 Quantitative analysis of all subjects

**FIGURE 5:**
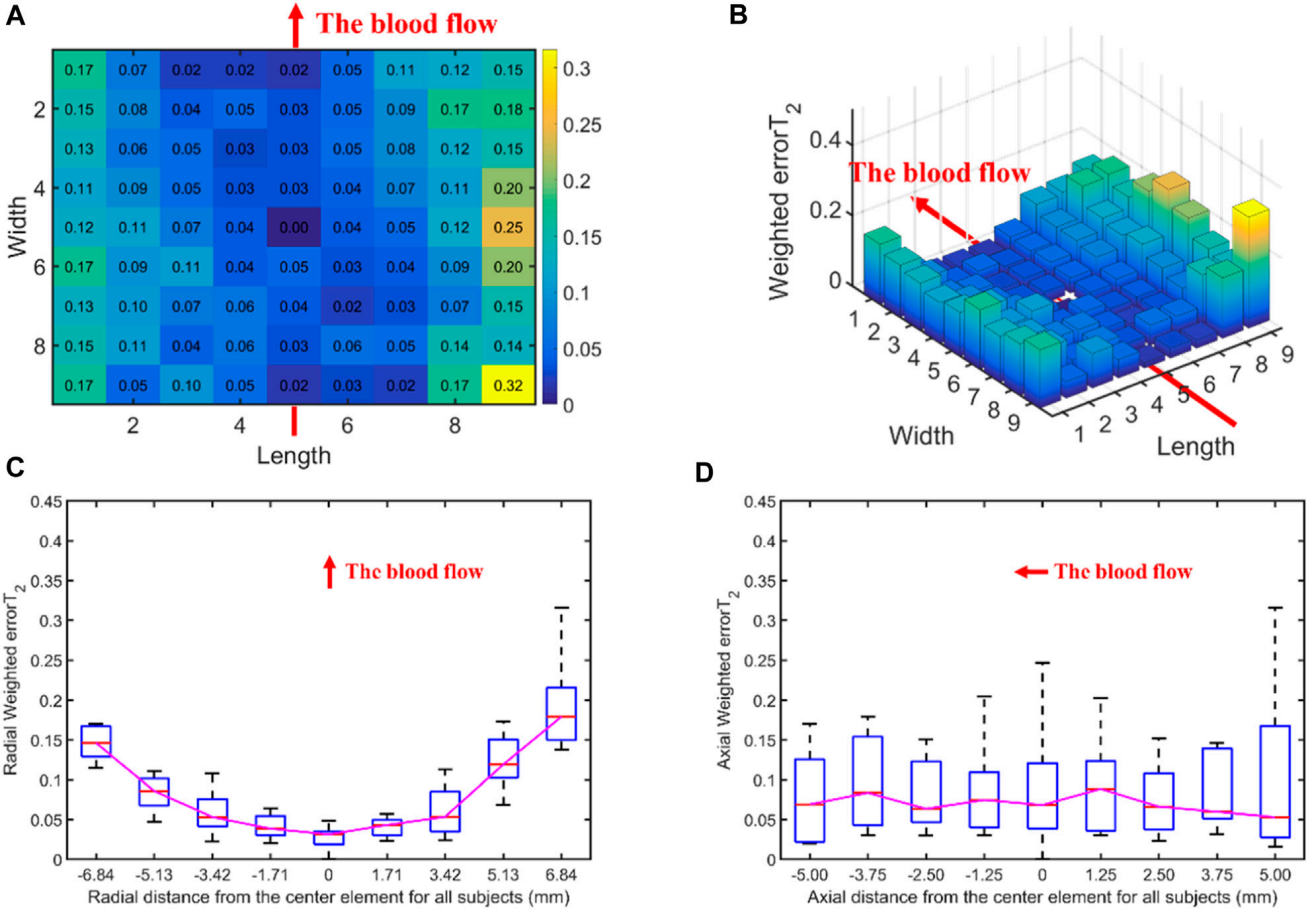
The errors between each channel and the best channel of all subjects: **(A)** The heat map of the errorT_2_SS for all subjects, with deeper colors indicating higher frequencies and larger error values; **(B)** The 3D bar chart of the errorT_2_SS for all subjects, where the *z*-axis represents the errorT_2_ values; **(C)** The radial weighted errorT_2_–distance boxplot of all subjects, *x*-axis represents the distance between sensing elements and center sensing elements, *y*-axis represents the radial weighted errorT_2_, a unit of the *x*-axis represents 1.71 mm; **(D)** The axial weighted errorT_2_–distance boxplot of all subjects, a unit of the *x*-axis represents 1.25 mm. A sensing element is a channel in a sensor. The red arrow indicates the direction of blood flow.

Figure 5 displays the errorT_2_SS and weighted errorT_2_–distance relation for all subjects. Figures 5A, B present the heat map and 3D bar of the errorT_2_SS for all subjects, respectively. The errorT_2_SS demonstrates a central low and peripheral high pattern. The further away from the center point, the higher the weighted errorT_2_ trend. Furthermore, an upward trend is observed at both ends of the axis corresponding to the blood flow direction, with higher weighted errorT_2_ as distance from the vessel increases. The central value is 0, as it represents that the central channel covers the best channels of all samples. Figures 5C, D illustrate the weighted errorT_2_–distance boxplots for all subjects. The boxplots indicate nonlinear relationships between the increase in distance and the corresponding increase in errorT_2_. For every 1 mm increase in distances between sensing elements and center sensing elements, the weighted errorT_2_ in the radial direction escalates by 4.87%. The weighted errorT_2_ tends to be stable in the axial direction. As the distances from the center channel increase, the volatilities of the errorT_2_ become more pronounced. When the distance is greater than 3.42 mm, the radial weighted errorT_2_ experiences a sudden increase.

Table 2 shows the $\bar{errorT_{2}}$ on different control groups in this study. In summary, the $\bar{errorT_{2}}$ of all subjects is 0.058. The $\bar{errorT_{2}}$ for left-handed subjects and non-hypertensive subjects are relatively lower, at 0.053 and 0.052. In comparison, right-handed subjects, males, and hypertensive subjects exhibit higher $\bar{errorT_{2}}$ of 0.065, 0.059, and 0.060, respectively. With regard to gender, male subjects have a slightly lower $\bar{errorT_{2}}$ than females, with values of 0.059 and 0.057.

**TABLE 2:** $\bar{errorT_{2}}$ on different control groups.

	$\bar{errorT_{2}}$
All subjects	0.058 ± 0.061
Left hands	0.053 ± 0.052
Right hands	0.065 ± 0.068
Male	0.059 ± 0.060
Female	0.057 ± 0.067
Non-hypertensive subjects	0.052 ± 0.048
Hypertensive subjects	0.060 ± 0.063

### 3.2 Comparative study for all control groups

#### 3.2.1 Left hands vs. Right hands

**FIGURE 6:**
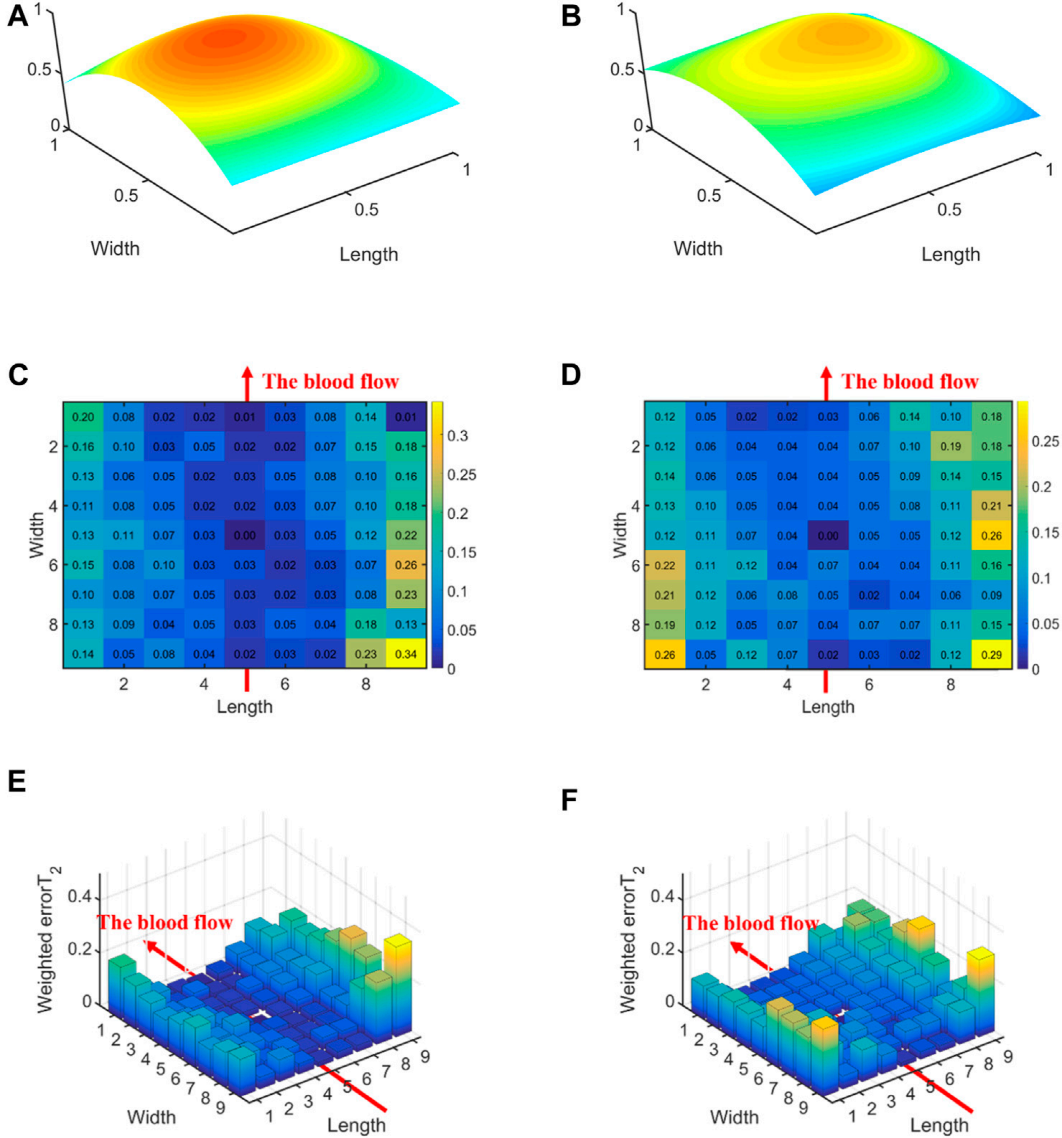
The errors between each channel and the best channel for left and right hands: **(A)** The normalized 3DPI in T_2_ of all samples of left hands; **(B)** The normalized 3DPI in T_2_ of all samples of right hands; **(C)** The heat map of errorT_2_SS of all samples of left hands; **(D)** The heat map of errorT_2_SS of all samples of right hands; **(E)** The 3D bar of errorT_2_SS of all samples of left hands; **(F)** The 3D bar of errorT_2_SS of all samples of right hands. The red arrow indicates the direction of blood flow.

**FIGURE 7:**
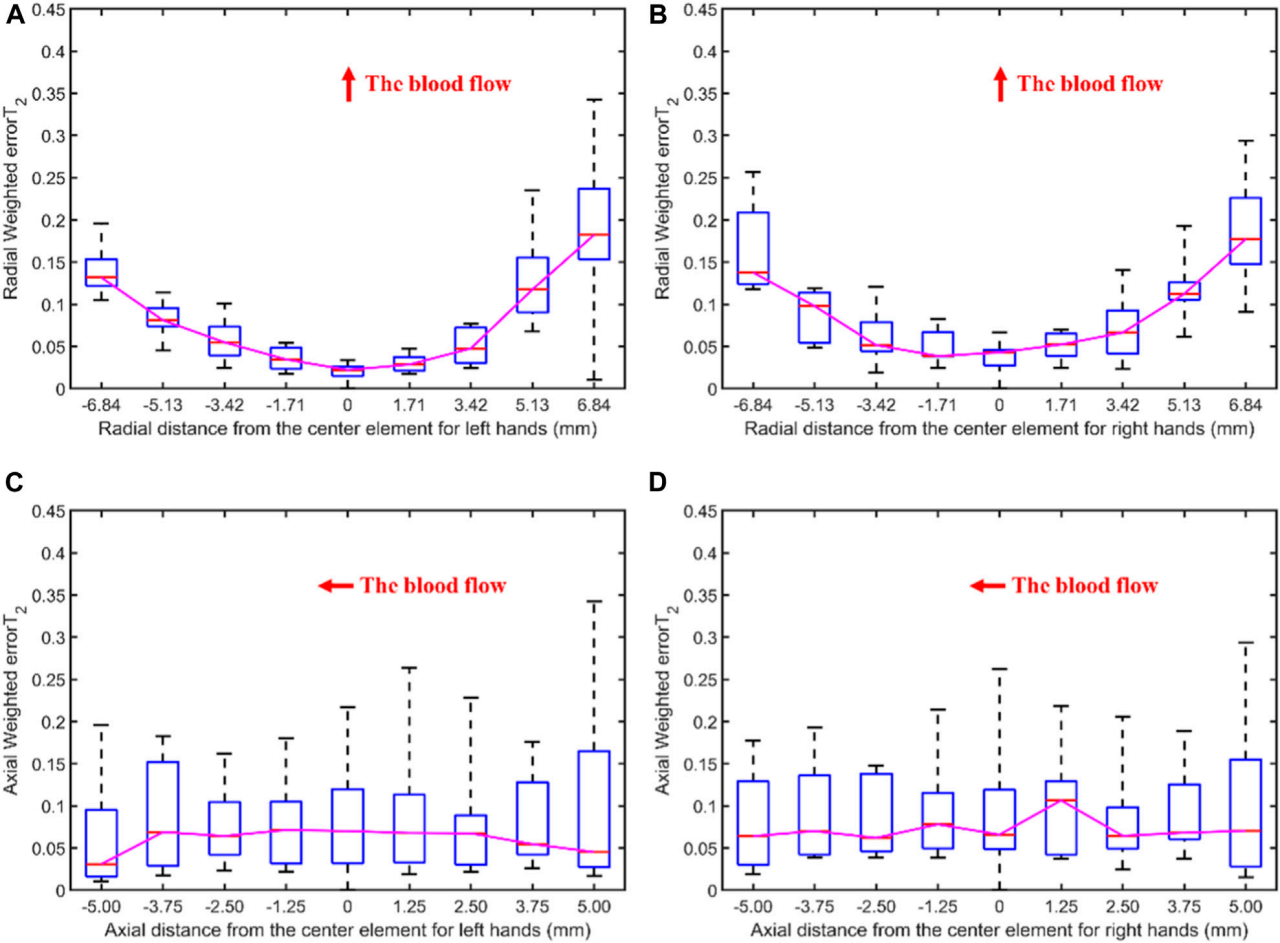
The weighted errorT_2_–distance boxplots for left and right hands: **(A)** The radial weighted errorT_2_–distance boxplot of left hands, *x*-axis represents the distance between sensing elements and center sensing elements, *y*-axis represents the radial weighted errorT_2_, a unit of the *x*-axis represents 1.71 mm; **(B)** The radial weighted errorT_2_–distance boxplot of right hands, a unit of the *x*-axis represents 1.71 mm; **(C)** The axial weighted errorT_2_–distance boxplot of left hands, a unit of the *x*-axis represents 1.25 mm; **(D)** The axial weighted errorT_2_–distance boxplot of right hands, a unit of the *x*-axis represents 1.25 mm. A sensing element is a channel in a sensor. The red arrow indicates the direction of blood flow.

Figure 6 displays the errors between each channel and the best channel of left and right hands. The errorT_2_SS exhibits a central low and peripheral high pattern for both hands. Moreover, an upward trend is observed at both ends of the axis corresponding to the blood flow direction. It can be observed that the error distributions for both hands show a subtle difference compared to the average error (Figure 5) distribution for all subjects. The boxplots in Figure 7 indicate that for every 1 mm increase in distance, the weighted errorT_2_ in the radial direction for left and right hands escalate by 4.55% and 5.04%, respectively. When the distance is greater than 3.42 mm, the radial weighted errorT_2_ for the left and right hands experience sudden increases.

#### 3.2.2 Male vs. Female

**FIGURE 8:**
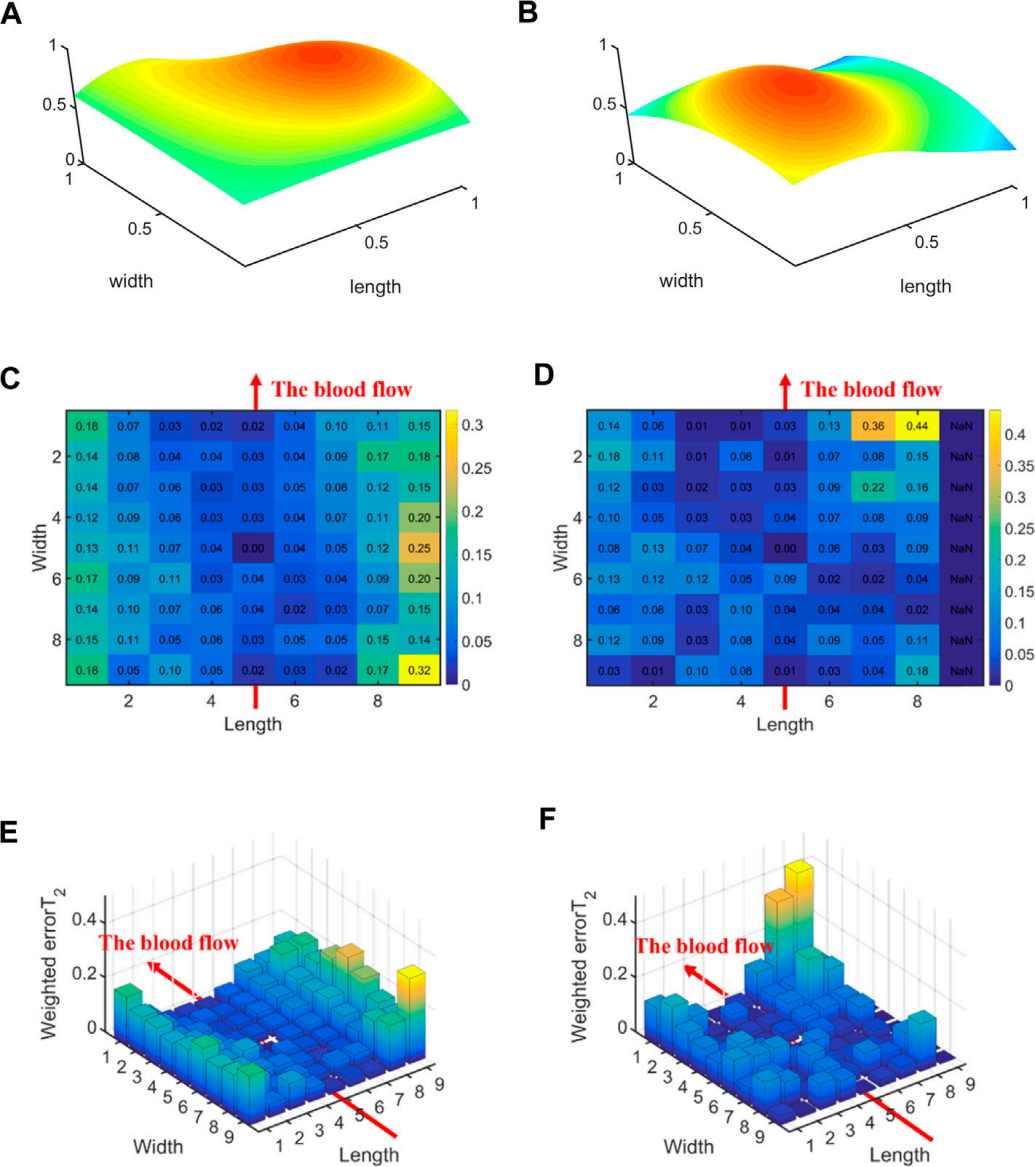
The errors between each channel and the best channel for male and female subjects: **(A)** The normalized 3DPI in T_2_ of all samples of male subjects; **(B)** The normalized 3DPI in T_2_ of all samples of female subjects; **(C)** The heat map of errorT_2_SS of all samples of male subjects; **(D)** The heat map of all samples of female subjects; **(E)** The 3D bar of errorT_2_SS of all samples of male subjects; **(F)** The 3D bar of errorT_2_SS of all samples of female subjects. The red arrow indicates the direction of blood flow.

**FIGURE 9:**
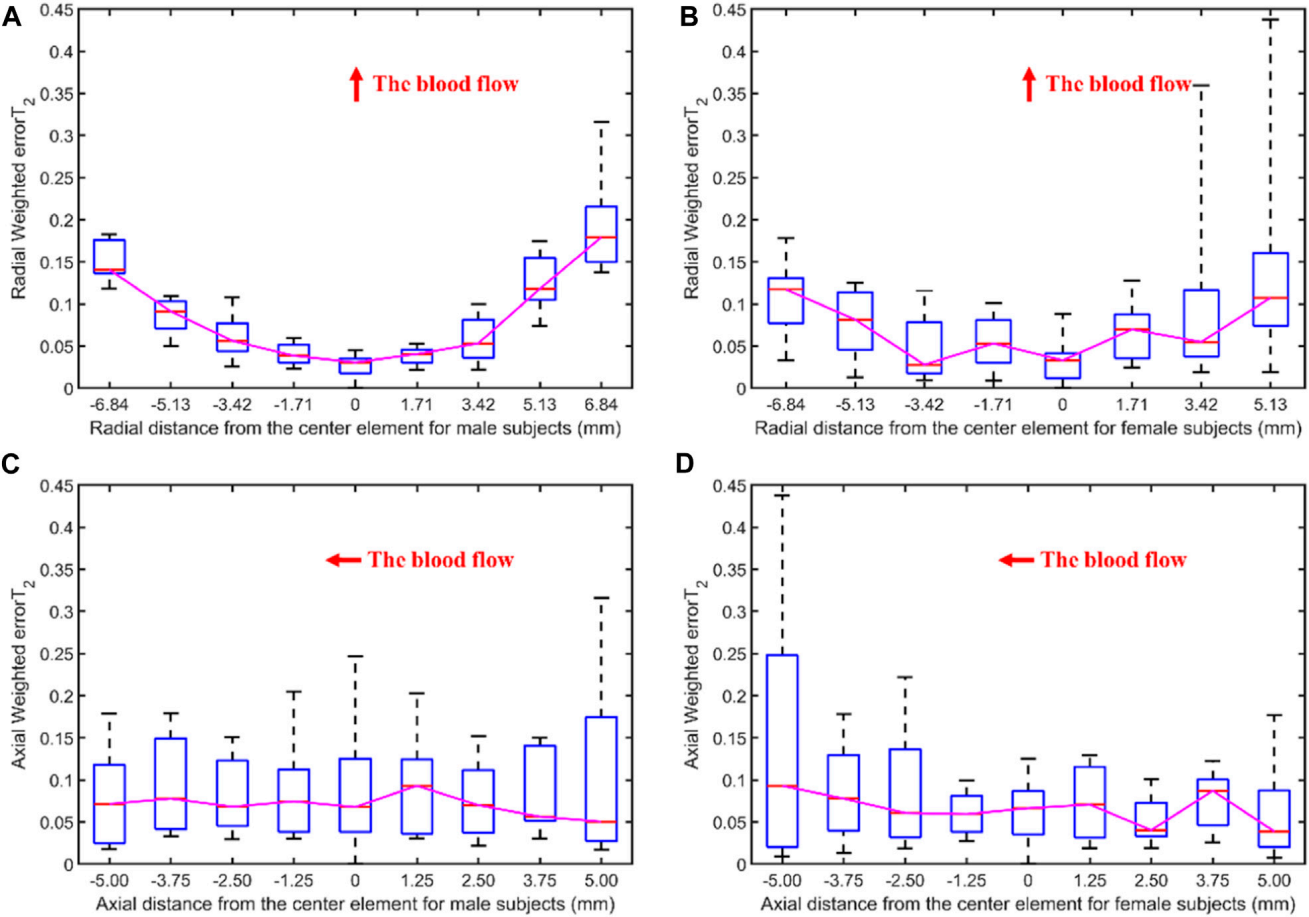
The weighted errorT_2_–distance boxplots for male and female subjects: **(A)** The radial weighted errorT_2_–distance boxplot of male subjects, *x*-axis represents the distance between sensing elements and center sensing elements, *y*-axis represents the radial weighted errorT_2_, a unit of the *x*-axis represents 1.71 mm; **(B)** The radial weighted errorT_2_–distance boxplot of female subjects, a unit of the *x*-axis represents 1.71 mm; **(C)** The axial weighted errorT_2_–distance boxplot of male subjects, a unit of the *x*-axis represents 1.25 mm; **(D)** The axial weighted errorT_2_–distance boxplot of female subjects, a unit of the *x*-axis represents 1.25 mm. A sensing element is a channel in a sensor. The red arrow indicates the direction of blood flow.

Figure 8 illustrates the errors between each channel and the best channel of male and female subjects. The errorT_2_SS shows a central low and peripheral high pattern for both male and female, with an upward trend at both ends of the axis corresponding to blood flow direction. It is noteworthy that the right-most channels of the errorT_2_SS for female samples exhibit data missing, suggesting that no channels were stacked on the base channels during the stacking of errorT_2_S for female subjects. The weighted errorT_2_ in the upper-right corner of the errorT_2_SS for females are relatively greater, which may be caused by outlier values. Figure 9 shows that for every 1 mm increase in distance, the weighted errorT_2_ in the radial direction for male and female subjects rise by 4.86% and 3.98%, respectively. When the distance is greater than 3.42 mm, the radial weighted errorT_2_ for the male subjects experiences a sudden increase. Since the data in the right-most channels is missing, there are only 8 distance groups in Figure 9B.

#### 3.2.3 Non-hypertensive vs. Hypertensive

**FIGURE 10:**
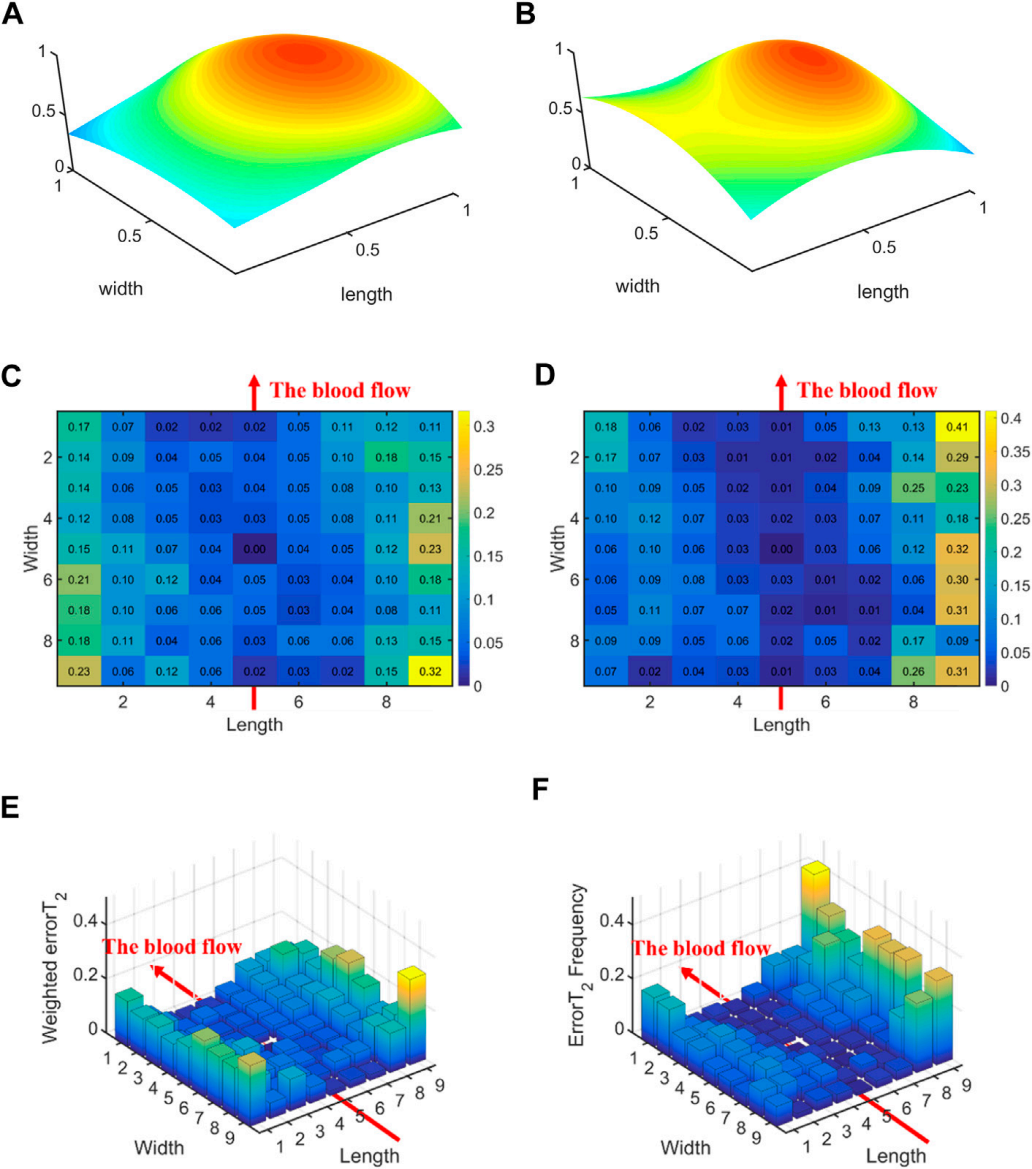
The errors between each channel and the best channel for non-hypertensive and hypertension subjects: **(A)** The normalized 3DPI in T_2_ of all samples of non-hypertensive subjects; **(B)** The normalized 3DPI in T_2_ of all samples of hypertension subjects; **(C)** The heat map of errorT_2_SS of all samples of non-hypertensive subjects; **(D)** The heat map of errorT_2_SS of all samples of hypertensive subjects; **(E)** The 3D bar of errorT_2_SS of all samples of non-hypertensive subjects; **(F)** The 3D bar of errorT_2_SS of all samples of hypertensive subjects. The red arrow indicates the direction of blood flow.

**FIGURE 11:**
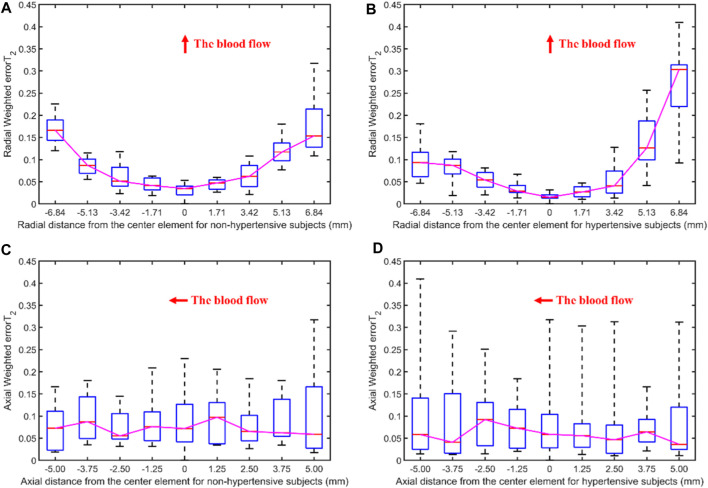
The weighted errorT_2_–distance boxplots for non-hypertensive and hypertensive subjects: **(A)** The radial weighted errorT_2_–distance boxplot of non-hypertensive subjects, *x*-axis represents the distance between sensing elements and center sensing elements, *y*-axis represents the radial weighted errorT_2_, a unit of the *x*-axis represents 1.71 mm; **(B)** The radial weighted errorT_2_–distance boxplot of hypertensive subjects, a unit of the *x*-axis represents 1.71 mm; **(C)** The axial weighted errorT_2_–distance boxplot of non-hypertensive subjects, a unit of the *x*-axis represents 1.25 mm; **(D)** The axial weighted errorT_2_–distance boxplot of non-hypertensive subjects, a unit of the *x*-axis represents 1.25 mm. A sensing element is a channel in a sensor. The red arrow indicates the direction of blood flow.

Figure 10 displays the errors between each channel and the best channel for non-hypertensive and hypertensive subjects. The errorT_2_SS exhibits a pattern of central decrement and peripheral increment, with an ascending trend at both extremities of the axis corresponding to the direction of blood flow. It can be inferred that the errorT_2_SS for non-hypertensive subjects is more evenly distributed compared to hypertensive patients overall, whereas the errorT_2_SS for hypertensive patients exhibits smaller errors along the arterial axis. Figure 11 indicates that for every 1 mm increase in distance, the weighted errorT_2_ in the radial direction for non-hypertensive and hypertension subjects rise by 4.94% and 5.04%, respectively. When the distance is greater than 3.42 mm, the radial weighted errorT_2_ for the non-hypertensive and hypertension subjects experience sudden increases.

## 4 Discussion

This study discovers the pressing offsets in multi-channel pulse signals and analyzes the relationship between the pressing offsets and T_2_ by qualifying the pressing offsets in pulse signal acquisition. First, we employ a data acquisition system to obtain 3DPIs from the subjects. Subsequently, the errors between each channel and the best channel are determined. The error T_2_ Stacked Surface (errorT_2_SS) and the average error T_2_ ( $\bar{errorT_{2}}$ ) are implemented to qualify the pressing offsets in the tactile sensor. Finally, the subjects are divided into multiple control groups, and the results are compared and analyzed.

Figures 5, 6, 8, 10 display the error between each channel and the best channel for all control groups. All errorT_2_SS in these figures exhibit a central low and peripheral high pattern. In each errorT_2_SS, the further away from the central point, the more upward the trend for all channels. The comparative study shows similar patterns for all subjects and control groups, revealing that our qualifications meet the true conditions of arterial pulse signals and the characteristics of the human radial arteries. The center of each errorT_2_SS reflects the offsets of the best channels, which are the smallest among all channels. The positions of the best channels align with the center of pressure on the arteries. Furthermore, the error values increased as distance from the artery increased, particularly at the axial ends of the blood flow direction. In Figure 8, data missing is existed in the right-most column of the errorT_2_SS for female subjects. Considering the sample size of female subjects in this study, the errorT_2_S of female samples may not thoroughly cover the entire 9 × 9 base matrix. Another possible reason is that the arteries of women exhibit more diminutive diameters (Xu et al., 2017; Deora et al., 2022) and are more difficult to adapt to the robotic fingertips. The inspiration from the results is selecting sensors with tiny sensing elements for pulse signal acquisition for female subjects. Figure 10 shows that the error distribution of non-hypertensive subjects is more uniform compared to hypertensive patients, while the errorT_2_SS of hypertensive patients has lower errors in the arterial axis. Compared to hypertensive patients, non-hypertensive subjects have lower vascular stiffness (Su et al., 2016; Zhang et al., 2021). Subsequently, during the pressing process of the robotic finger, the offsets might occur less.

The boxplots in Figures 7, 9, 11 indicate nonlinear relationships between the increase in distance and the corresponding increase in errorT_2_. For every 1 mm increase in distances between sensing elements and center sensing elements, the weighted errorT_2_ in the radial direction escalates by 4.87%. The weighted errorT_2_ escalations for left hands/right hands, male/female, and non-hypertensive/hypertensive are 4.55%/5.04%, 4.86%/3.98%, and 4.94%/5.04%, respectively. The clinical insights derived from these distinct results indicate that each clinical population possesses distinct physiological characteristics. Therefore, when acquiring pulse signals, it is essential to employ appropriate collection and measurement techniques to mitigate pressing offsets. For all control groups, when the distance is larger than 3.42 mm, the weighted radial errorT_2_ experience sudden increases for all subjects and control groups. This result can assist and guide operators in pressing processes. In clinical practice, we recommend the pressing offset of sensors not exceed 3.42 mm, which can adjust by qualifying the evaluation metrics. The weighted errorT_2_ tends to be stable in the axial direction. To our knowledge, the reason is that axial direction is the direction of the blood flow. The changes in the mechanical environment are different between axial and radial of the arterial smooth muscle (Messas et al., 2013; Rothermel et al., 2020).

When the robotic fingers of pulse acquisition devices apply pressing, offset may occur, potentially affecting measurement outcomes. In scenarios involving single or few-channel sensors, such offsets have more substantial impacts on measurement results. In such cases, the operators may face difficulties in establishing whether the pressing process is accompanied by offsets. Such hindrance could potentially detract physicians from making accurate assessments of cardiovascular conditions in patients. This study detects pressing offsets in radial arterial pulse signals and explores the solution to prevent pressing offsets. In the analysis of this study, the errorT_2_ enables the quantification and evaluation of 3DPI, thus allowing for the determination of the offset degree during the pressing process and subsequent correction. With adequate sensor channels, we can implement the measurements in this study to qualify and evaluate the pressing offsets. However, when the number of sensor channels is one or few, it is hard to use the measurements to determine the pressing offset, and the operators face difficulties in establishing whether the pressing process is accompanied by offsets. Furthermore, only adequate sensor channels can help operators obtain more accurate clinical and physiological parameters, which are essential for the further assessment and diagnosis of physiological conditions in subjects. This is undoubtedly of great importance in clinical practice. Therefore, we highlight that increasing the sensor channels is crucial for achieving quantitative offset assessment.

Furthermore, the arterial conditions are compared and analyzed under different circumstances. This study employs three control groups: left hands/right hands, male/female, and non-hypertensive/hypertensive subjects. During the pressing process, the tactile sensors of the pulse signal acquisition instrument are similarly influenced by the arteries of both hands. In contrast, the non-hypertensive/hypertensive subjects control group demonstrates a higher arterial stiffness in the hypertensive subjects, which readily affects the pressing process of the robotic finger (Milkovich et al., 2022). However, when the number of channels is limited to a single or few, analyzing these situations becomes challenging. Consequently, the most crucial aspect for analyzing the arterial characteristics of the subjects through sensors is increasing the sensor channels.

## 5 Conclusion

In scenarios involving single or few-channel sensors, pressing offsets have substantial impacts on measurement results. By detecting whether there are any offsets occurring during the pressing process, operators can adjust the pressing position to ensure the acquisition of 3DPIs that accurately reflect the arterial conditions. This study discovers the pressing offsets in multi-channel pulse signals and analyzes the relationship between the pressing offsets and T_2_ using a method to qualify the pressing offsets in pulse signal acquisition. First, we design a data acquisition system to capture 3DPIs from the subjects. Second, the errorT_2_ Stacked Surface (errorT_2_SS) and the average error T_2_ ( $\bar{errorT_{2}}$ ) are proposed to qualify the pressing offsets in the tactile sensors. Finally, the subjects are divided into several control groups for subsequent analysis. The comparative study for errorT_2_SS exhibits a central low and peripheral high pattern. Furthermore, the error values increased as distance from the artery increased, particularly at the axial ends of the blood flow direction. For every 1 mm increase in distances between sensing elements and center sensing elements, the weighted errorT_2_ in the radial direction escalates by 4.87%. When the distance is greater than 3.42 mm, the weighted radial errorT_2_ experience sudden increases for all subjects and control groups. The weighted errorT_2_ tends to be stable in the axial direction. The subjects in this study are middle-aged and elderly subjects aged 40–80 years, leading to a potential lack of representativeness. In the future, several enhancements may be contemplated for application in subsequent research. In the end, it remains imperative to highlight the necessity of increasing the sensor channels.

## 6 Limitation

Although this study discovers pressing offsets in radial pulse signal acquisition, some limitations should be reported. The subjects in this study are middle-aged and elderly subjects aged 40–80 years, leading to a potential lack of representativeness. The subjects aged 40–80 years may hinder the generalization of the methods presented in this study to younger subjects. In addition, the presence of missing data in errorT_2_SS for female subjects is due to the weighted errorT_2_ absence of these regions, which may potentially affect the analysis of pressing offsets in female subjects, thereby hindering the acquisition of accurate conclusions. More young subjects and female subjects should be included. In terms of prospects, several enhancements may be contemplated for application in subsequent research. For instance, broaden the age range of the sample population and include more control groups, such as subjects with other diseases, before and after exercise, and different postures, to make the research more universally applicable to the physical characteristics of blood vessels. In addition to expanding the composition of the sample, incorporating real-time measurement adjustments based on pressing offsets could serve as a potential future research enhancement.

## Data Availability

The raw data supporting the conclusion of this article will be made available by the authors, without undue reservation.
