# Corrigendum: Increasing the sensor channels: a solution for the pressing offsets that cause the physiological parameter inaccuracy in radial artery pulse signal acquisition

**DOI:** 10.3389/fbioe.2024.1487592

**Published:** 2024-10-21

**Authors:** Chao Chen, Zhendong Chen, Hongmiin Luo, Bo Peng, Yinan Hao, Xiaohua Xie, Haiqing Xie, Xinxin Li

**Affiliations:** ^1^ School of Computer Science and Engineering, Sun Yat-Sen University, Guangzhou, China; ^2^ Science and Technology Innovation Center, Guangzhou University of Chinese Medicine, Guangzhou, China; ^3^ Department of Musical Instrument Engineering, Xinghai Conservatory of Music, Guangzhou, China; ^4^ Sniow Research and Development Laboratory, Foshan, China; ^5^ School of Medical Engineering, Foshan University, Foshan, China; ^6^ School of Nursing, Sun Yat-Sen University, Guangzhou, China

**Keywords:** multi-channel pulse signals, tactile sensors, tidal peak, pulse wave analysis, biomedical engineering

In the published article, there was an error in Figure 8A as published. During the layout process of the manuscript, the figure is inadvertently overwritten by **Figure 6A**. The corrected Figure 8A and its caption *The normalized 3DPI in T2 of all samples of male subjects* appear below.

**FIGURE 8:**
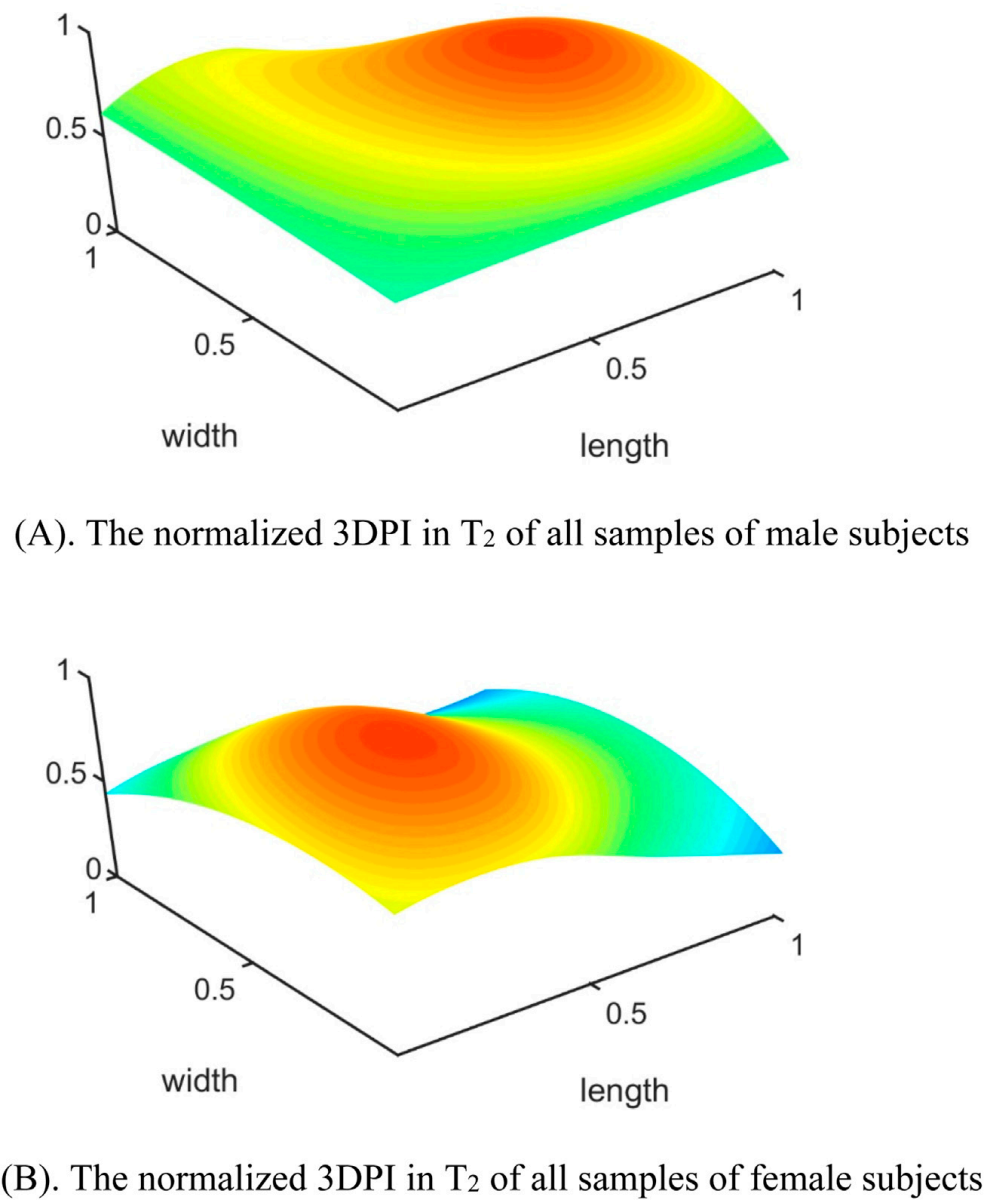
The errors between each channel and the best channel for male and female subjects: **(A)** The normalized 3DPI in T_2_ of all samples of male subjects; **(B)** The normalized 3DPI in T_2_ of all samples of female subjects; **(C)** The heat map of errorT_2_SS of all samples of male subjects; **(D)** The heat map of all samples of female subjects; **(E)** The 3D bar of errorT_2_SS of all samples of male subjects; **(F)** The 3D bar of errorT_2_SS of all samples of female subjects. The red arrow indicates the direction of blood flow.

In the published article, there was an error in Figure 8B as published. During the layout process of the manuscript, the figure is inadvertently overwritten by **Figure 6B**. The corrected Figure 8B and its caption *The normalized 3DPI in T2 of all samples of female subjects* appear below.

In the published article, there was an error in Figure 10A as published. During the layout process of the manuscript, the figure is inadvertently overwritten by **Figure 6A**. The corrected Figure 10A and its caption *The normalized 3DPI in T2 of all samples of non-hypertensive subjects* appear below.

**FIGURE 10:**
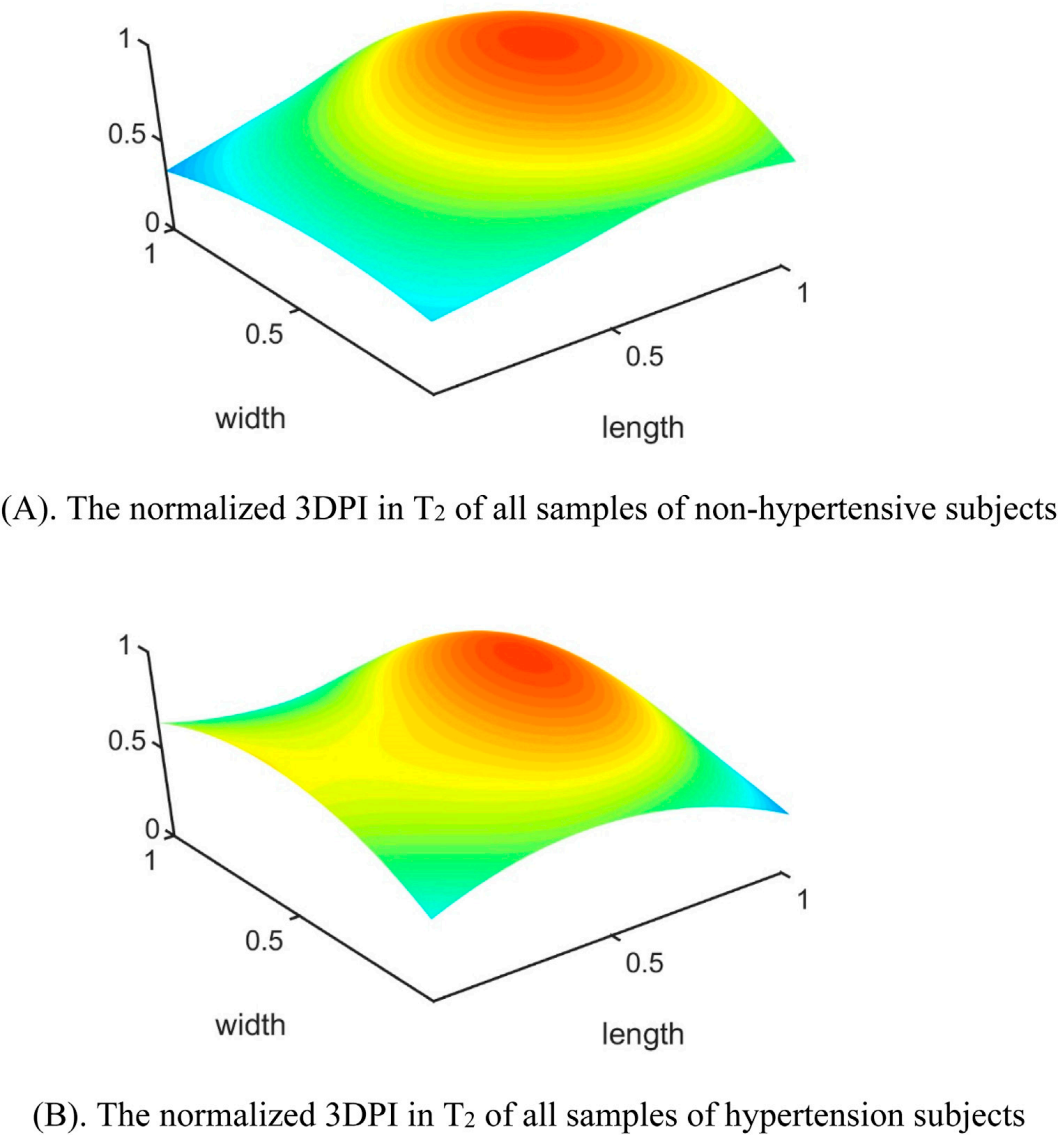
The errors between each channel and the best channel for non-hypertensive and hypertension subjects: **(A)** The normalized 3DPI in T_2_ of all samples of non-hypertensive subjects; **(B)** The normalized 3DPI in T_2_ of all samples of hypertension subjects; **(C)** The heat map of errorT2SS of all samples of non-hypertensive subjects; **(D)** The heat map of errorT2SS of all samples of hypertensive subjects; **(E)** The 3D bar of errorT2SS of all samples of non-hypertensive subjects; **(F)** The 3D bar of errorT2SS of all samples of hypertensive subjects. The red arrow indicates the direction of blood flow.

In the published article, there was an error in Figure 10B as published. During the layout process of the manuscript, the figure is inadvertently overwritten by **Figure 6B**. The corrected Figure 10B and its caption *The normalized 3DPI in T2 of all samples of hypertension subjects* appear below.

The authors apologize for this error and state that this does not change the scientific conclusions of the article in any way. The original article has been updated.

